# Adiposity Markers as Predictors of 11-Year Decline in Maximal Walking Speed in Late Midlife

**DOI:** 10.1177/0733464820911542

**Published:** 2020-03-13

**Authors:** Heini Wennman, Gerald J. Jerome, Eleanor M. Simonsick, Päivi Sainio, Heli Valkeinen, Katja Borodulin, Sari Stenholm

**Affiliations:** 1 Finnish Institute for Health and Welfare, Helsinki, Finland; 2 Towson University, MD, USA; 3 National Institute on Aging, Baltimore, MD, USA; 4 Age Institute, Helsinki, Finland; 5 University of Turku, Finland; 6 Turku University Hospital, Finland

**Keywords:** obesity, body mass index, waist circumference, walking speed, midlife, mobility

## Abstract

**Background:** Obesity is linked to poorer physical functioning in older adults, but impact of excess adiposity on loss of functional capacity in late midlife is unclear. This study examined associations between adiposity markers and 11-year change in maximal walking speed, a sensitive indicator of physical functioning, among adults aged 55 to 69 years. **Method:** Maximal walking speed over 6.1 m was assessed in 2000 and 2011 among Finnish men (*n* = 409) and women (*n* = 498) from the prospective Health 2000 Survey. Body mass index (BMI) and waist circumference were assessed in 2000. Generalized estimating equation models estimated changes in maximal walking speed by BMI and waist circumference, stratified by sex. **Results:** BMI greater than 30 kg/m^2^ was associated with accelerated decline in maximal walking speed particularly in women. Associations with waist circumference were nonsignificant. **Conclusion:** Late midlife obesity may speed up the decline in functional capacity as measured by maximal walking speed, especially in women.

## Introduction

Although adiposity is known to contribute to mobility decline in older adults, the impact of adiposity on mobility decline in middle-age and early old age is not as well-studied. Two previous long-term follow-up studies among initially middle-aged persons found that obesity (body mass index [BMI] > 30 kg/m^2^) predicted decline in usual walking speed and incidence of walking limitation (Stenholm, Sainio, et al., 2007; Windham et al., 2017). Limitations of these studies include consideration of only BMI as an indicator of adiposity and lack of sex-stratified analyses. Moreover, instead of using usual walking speed or self-reported information on walking limitation, it is of interest to examine changes in maximal walking speed, which is more sensitive marker of mobility decline than usual walking speed, as it often begins to decline for men in their fifties and women in their sixties (Ferrucci et al., 2016; Simonsick et al., 2005). In addition to mobility decline, a slower maximal walking speed strongly predicts decrease in functional capacity related to instrumental activities of daily life (Suzuki et al., 2003).

As both the number and proportion of late midlife adults with obesity have increased markedly over the past few decades, there is need to identify sensitive measures that would allow for early detection of mobility decline and decreased functional capacity appropriate for use in clinical practice. This study aims to examine the association between adiposity markers and 11-year change in maximal walking speed among Finnish men and women in late midlife.

## Method

### Study Population

The initial assessment occurred in the Health 2000 Survey, a comprehensive nationwide health interview and examination survey carried out in Finland in 2000–2001 with a follow-up measurement in 2011. Details of the design and implementation of the Health 2000 Survey and its follow-up have been reported elsewhere (Aromaa & Koskinen, 2004; Koskinen et al., 2012).

Inclusion criteria for this study were age 55 to 69 years, BMI ≥ 18.5 kg/m^2^, measured waist circumference and walking speed assessment without an assistive walking device at baseline. Of the 1,513 eligible participants in 2000, 980 (65%) participated in the follow-up health examination in 2011 and 930 (61%) completed follow-up walking speed assessment. An additional 23 subjects were excluded because of missing data on baseline covariates yielding an analytic sample of 907: 409 men and 498 women. Written informed consent was obtained from all individual participants included in the study.

### Measures

#### Adiposity markers

At baseline, height and weight were assessed in light clothing and without shoes. BMI was classified as normal-to-overweight (18.5–29.9 kg/m^2^) and obese (≥30 kg/m^2^) (World Health Organization, 2000). Established cutoffs identified high waist circumference in women (>88 cm) and men (>102 cm) with lower values considered normal (World Health Organization, 2000).

#### Maximal walking speed

At baseline, and 11-year follow-up, participants walked 6.1 m as fast as possible, starting from a standing position, continuing at a maximum pace over the finish line (Sainio et al., 2008).

#### Covariates

All covariates were evaluated at baseline. Grip strength was assessed as the higher of two measurements on the dominant hand (Sainio et al., 2008). Self-reported leisure-time physical activity was classified into three categories based on the volume of activity: low, medium, and high (Borodulin et al., 2016). Information on smoking, alcohol consumption, and baseline education were self-reported using a standardized questionnaire (Aromaa & Koskinen, 2004).

### Statistical Analyses

The differences in background characteristics by sex were explored by chi-square test for categorical variables and analysis of variance or Wilcoxon Scores test for continuous variables. Normality of maximal walking speed was verified. The association between BMI (normal-to-overweight or obese) and waist circumference (low or high) with change in maximal walking speed was examined using generalized estimating equation (GEE) models, stratified by sex, including either a BMI and time, or a waist circumference and time interaction term in the models. Analyses controlled for baseline education, smoking, alcohol consumption, leisure-time physical activity, and grip strength. Statistical analyses were performed using SAS version 9.3 (SAS Institute, Inc., Cary, NC).

## Results

Characteristics of the study population are presented in Table 1. Participants excluded from the analyses due to missing 11-year follow-up data were older and had a slower walking speed, higher waist circumference, lower education levels, smoked more often, and were less physically active in 2000 as compared with those included in the analysis (all *p* < .05).

**Table 1.** Baseline Characteristics of the Study Population.

Variables	Men (*n* = 409)	Women (*n* = 498)	*p*-value
	*M* (*SD*)	*M* (*SD*)	
Age (years)	61 (4)	61 (4)	.738
Maximal walking speed (m/s)	1.84 (0.34)	1.58 (0.28)	<.001
Grip strength (N)	465 (84)	262 (64)	<.001
	*n* (%)	*n* (%)	
Body mass index			.06
Normal to overweight (18.5–29.9 kg/m^2^)	312 (76)	352 (71)	
Obese (≥30 kg/m^2^)	97 (24)	146 (29)	
Waist circumference			<.001
Low (men ≤ 102 cm, women ≤ 88 cm)	258 (63)	232 (47)	
High (men > 102 cm, women > 88 cm)	151 (37)	266 (53)	
Education			.06
Basic	193 (47)	260 (52)	
Intermediate	127 (31)	119 (24)	
High	89 (22)	119 (24)	
Smoking			<.001
Current/occasional	73 (18)	57 (11)	
Former	170 (42)	60 (12)	
Never/nonsmoker more than 1 year	166 (41)	381 (77)	
Alcohol			<.001
Men < 7 or Women < 4 (portions/week)	271 (66)	406 (82)	
Men ≥ 7 or Women ≥ 4 (portions/week)	138 (34)	92 (18)	
Physical activity			<.001
Low	64 (16)	96 (19)	
Moderate	247 (60)	339 (68)	
High	98 (24)	63 (13)	

*Note. p*-value for comparison between sexes by chi-square, ANOVA, or Wilcoxon Scores test. ANOVA = analysis of variance.

Among the 907 subjects remaining in the sample, at baseline the mean maximal walking speed was 1.84 m/s (95% confidence interval [CI] = [1.81, 1.88]) in men and 1.58 m/s (95% CI = [1.55, 1.60]) in women. Walking speed declined significantly over 11 years in men (−0.22 m/s, 95% CI = [−0.26, −0.18]) and in women (−0.16 m/s, 95% CI = [−0.18, −0.13]).

**Figure 1.**
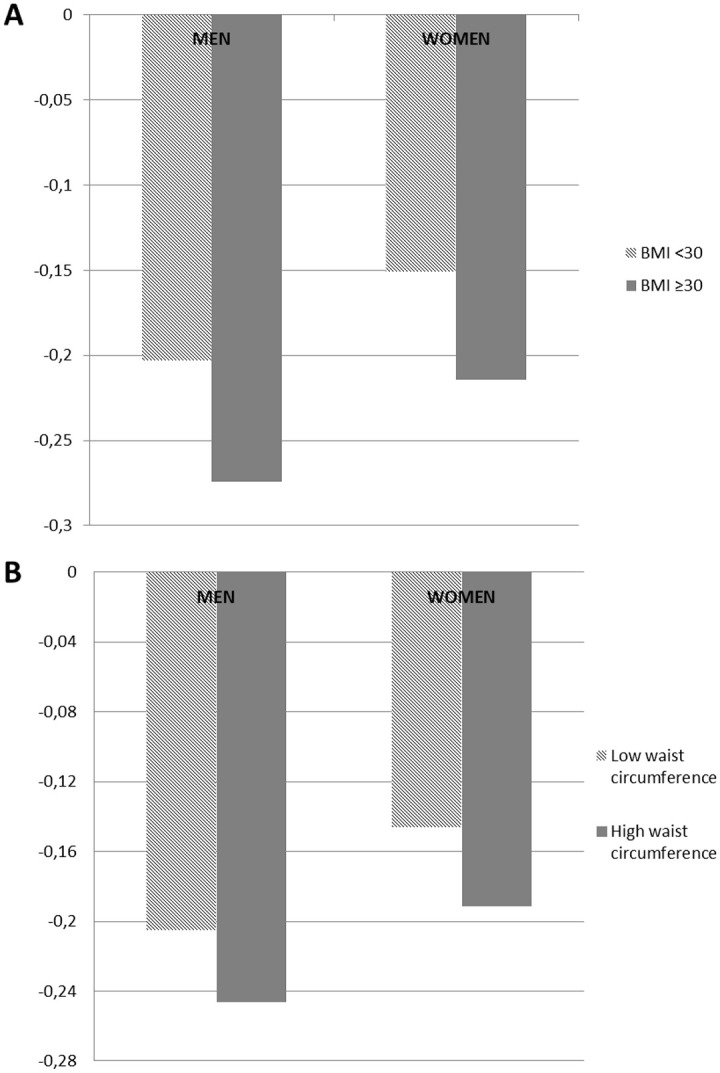
Eleven-year decline in maximal walking speed for men and women by BMI (A) and by waist circumference (B). *Note.* BMI = body mass index.

The average decline in maximal walking speed for men and women by adiposity markers is presented in Figure 1 and Table 2. The 11-year decline in maximal walking speed was significantly greater in the obese women than in the normal-to-overweight women (−0.21 m/s vs. −0.15 m/s, *p* for BMI × Time interaction = .037). No difference in maximal walking speed decline by obesity group was observed for men (Table 2). The decline in maximal walking speed did not differ by waist circumference groups in either women or men.

**Table 2.** Baseline Maximal Walking Speed (m/s) and 11-Year Change (m/s) by BMI and Waist Circumference in Men and Women Aged 55 to 69 Years.

Variables	Men (*n* = 409)		Women (*n* = 498)	
	Baseline maximal walking speed	11-year change	Baseline maximal walking speed	11-year change
BMI				
<30	1.85 [1.81, 1.90]	−0.20 [−0.25, −0.16]	1.60 [1.56, 1.65]	−0.15 [−0.18, −0.12]
≥30	1.80 [1.73, 1.88]	−0.27 [−0.36, −0.19]	1.51 [1.46, 1.56]	−0.21 [−0.26, −0.17]
BMI × Time		*p* = .135		*p* = .037
Waist circumference				
Low	1.89 [1.84, 1.93]	−0.21 [−0.25, −0.16]	1.59 [1.55, 1.64]	−0.15 [−0.19, −0.10]
High	1.77 [1.72, 1.82]	−0.25 [−0.31, −0.18]	1.56 [1.52, 1.61]	−0.19 [−0.23, −0.16]
Waist × Time		*p* = .309		*p* = .101

*Note.* Analyses adjusted for age, education, smoking, alcohol, physical activity, and grip strength. BMI = body mass index.

## Discussion

In a sample of late middle-aged Finnish adults, a significant decline in maximal walking speed across 11 years was observed. A greater 11-year decline in maximal walking speed was found in obese relative to normal-to-overweight women.

Although numerous studies have included maximal walking speed in their protocol, few published reports have examined maximal walking speed within sex and adiposity subgroups or investigated long-term decline. The baseline mean maximal walking speeds within BMI groups in this study were comparable with the 1.76 m/s (normal weight) and 1.69 m/s (obese) maximal walking speed observed for high functioning but slightly older (50–84 years) participants in the Baltimore Longitudinal Study of Aging (Ko et al., 2010).

The only study to our knowledge that examined maximal walking speed change over several years focused on nondisabled French men and women aged 65 to 85 years (Artaud et al., 2015). The average annual decline was −0.017 m/s which is comparable with, or somewhat lower than the annual decline observed in the current study in the obese women and men (range from −0.017 and −0.025 m/s per year).

The current finding is consistent with previous literature documenting the detrimental impact of obesity as assessed by BMI on functional decline in both older age and late midlife (Jerome et al., 2016; Stenholm et al., 2009; Stenholm, Rantanen et al., 2007; Stenholm, Sainio, et al., 2007; Windham et al., 2017). A history of obesity (BMI > 30 kg/m^2^) in midlife has been observed to associate with walking limitations (Stenholm, Rantanen, et al., 2007) and slower usual walking speed (Ferrucci et al., 2016), even several years later. Our findings extend previous findings by showing a negative effect of obesity on maximal walking speed development over 11 years.

It has been proposed that body composition, particularly higher body fat in women, accounts for differences in functional capacity between men and women at older age (Tseng et al., 2014). We did not find high waist circumference, as a marker of abdominal adiposity, to be related to the decline in maximal walking speed in either sex. Some have observed high waist circumference to associate with slower walking speed in older men but not older women (Tolea et al., 2010).

In this study, BMI and waist circumference were used as markers of adiposity, with standard clinical cut points for both for the comparability with other reports and to evaluate their clinical utility. There is some evidence however that these established cut points may not be optimal or may differ by sex as indicators of adiposity. For example, more than half of the women in the current sample had a high waist circumference by the definition that was used. A more specific marker of fat infiltration in muscle predicted decline in usual walking speed in both men and women (Beavers et al., 2013). The adiposity–function relationship is further complicated by underlying pathology that may result in unintentional weight loss (Harris, 2017).

As in many prospective studies, there was some drop out (35%) over the follow-up. Individuals who dropped out from the study had slower walking speed and less favorable sociodemographic and lifestyle profile (e.g., education, smoking, physical activity), but the prevalence of obese was similar to the included. As such, these results may present an optimistic view of maximal walking speed decline. To the extent selection bias was dependent on both functional capacity decline and adiposity, these analyses may underestimate the long-term impact of adiposity on maximal walking speed. This study utilized BMI as an indicator of general obesity and waist circumference as an indicator of abdominal obesity, but because both of these are indirect measures of adiposity, further study is warranted that uses other adiposity indicators such as fat mass.

Strengths of these analyses include the use of a national population-based sample with physical measurement of maximal walking speed, grip strength, and adiposity markers, and a long follow-up. The analyses also controlled for factors that may attenuate decline (physical activity and grip strength) helping us better understand the unique relation of adiposity to decline in walking speed.

## Conclusion

In conclusion, the findings highlight the importance of preventing and treating obesity in late middle-aged persons, as obesity was shown to accelerate decline in maximal walking speed and thereby contributes to premature loss of functional capacity with advancing age.

## References

[bibr1-0733464820911542] Aromaa A. Koskinen S. (Eds). (2004). Health and functional capacity in Finland: Baseline results of the Health 2000 health examination survey (B12/2004 ed.). Publications of the National Public Health Institute.

[bibr2-0733464820911542] Artaud F. Singh-Manoux A. Dugravot A. Tzourio C. Elbaz A. (2015). Decline in fast gait speed as a predictor of disability in older adults. Journal of the American Geriatrics Society, 63(6), 1129–1136. 10.1111/jgs.13442 26096387

[bibr3-0733464820911542] Beavers K. M. Beavers D. P. Houston D. K. Harris T. B. Hue T. F. Koster A. Newman A. B. Simonsick E. M. Studenski S. A. Nicklas B. J. Kritchevsky S. B. (2013). Associations between body composition and gait-speed decline: Results from the health, aging, and body composition study. The American Journal of Clinical Nutrition, 97(3), 552–560. 10.3945/ajcn.112.047860 23364001 PMC3578402

[bibr4-0733464820911542] Borodulin K. Harald K. Jousilahti P. Laatikainen T. Männistö S. Vartiainen E. (2016). Time trends in physical activity from 1982 to 2012 in Finland. Scandinavian Journal of Medicine & Science in Sports, 26(1), 93–100. 10.1111/sms.12401 25559167

[bibr5-0733464820911542] Ferrucci L. Cooper R. Shardell M. Simonsick E. M. Schrack J. A. Kuh D. (2016). Age-related change in mobility: Perspectives from life course epidemiology and geroscience. The Journals of Gerontology, Series A: Biological Sciences & Medical Sciences, 71(9), 1184–1194. 10.1093/gerona/glw043 PMC4978365 26975983

[bibr6-0733464820911542] Harris T. B. (2017). Weight and body mass index in old age: Do they still matter? Journal of the American Geriatrics Society, 65(9), 1898–1899. 10.1111/jgs.14952 28714125 PMC5704942

[bibr7-0733464820911542] Jerome G. J. Ko S. U. Chiles Shaffer N. S. Studenski S. A. Ferrucci L. Simonsick E. M. (2016). Cross-sectional and longitudinal associations between adiposity and walking endurance in adults age 60-79. The Journals of Gerontology, Series A: Biological Sciences & Medical Sciences, 71(12), 1661–1666. https://doi.org/glw054 10.1093/gerona/glw054 PMC5106854 26984392

[bibr8-0733464820911542] Ko S. Stenholm S. Ferrucci L. (2010). Characteristic gait patterns in older adults with obesity—Results from the Baltimore longitudinal study of aging. Journal of Biomechanics, 43(6), 1104–1110. 10.1016/j.jbiomech.2009.12.004 20080238 PMC2849896

[bibr9-0733464820911542] Koskinen S. Lundqvist A. Ristiluoma N. (Eds). (2012). Health, functional capacity and welfare in Finland in 2011 (Report 68/2012.). National Institute for Health and Welfare (THL). (In Finnish with English Abstract)

[bibr10-0733464820911542] Sainio P. Koskinen S. Natunen S. (2008). Functional capacity. In Heistaro S. (Ed.), Methodology report: Health 2000 survey. Publications of the National Public Health Institute. http://www.julkari.fi/handle/10024/78185

[bibr11-0733464820911542] Simonsick E. M. Guralnik J. M. Volpato S. Balfour J. Fried L. P. (2005). Just get out the door! Importance of walking outside the home for maintaining mobility: Findings from the women’s health and aging study. Journal of the American Geriatrics Society, 53(2), 198–203. https://doi.org/JGS53103 15673341 10.1111/j.1532-5415.2005.53103.x

[bibr12-0733464820911542] Stenholm S. Alley D. Bandinelli S. Griswold M. E. Koskinen S. Rantanen T. Guralnik J. M. Ferrucci L. (2009). The effect of obesity combined with low muscle strength on decline in mobility in older persons: Results from the InCHIANTI study. International Journal of Obesity, 33, 635–644. 10.1038/ijo.2009.62 19381155 PMC2697265

[bibr13-0733464820911542] Stenholm S. Rantanen T. Alanen E. Reunanen A. Sainio P. Koskinen S. (2007). Obesity history as a predictor of walking limitation at old age. Obesity, 15(4), 929–938. https://doi.org/15/4/929 17426328 10.1038/oby.2007.583

[bibr14-0733464820911542] Stenholm S. Sainio P. Rantanen T. Koskinen S. Jula A. Heliovaara M. Aromaa A. (2007). High body mass index and physical impairments as predictors of walking limitation 22 years later in adult Finns. The Journals of Gerontology, Series A: Biological Sciences & Medical Sciences, 62(8), 859–865. https://doi.org/62/8/859 10.1093/gerona/62.8.859 17702877

[bibr15-0733464820911542] Suzuki T. Yoshida H. Kim H. Yukawa H. Sugiura M. Furuna T. Nishizawa S. Kumagai S. Shinkai S. Ishizaki T. Watanabe S. Shibata H. (2003). Walking speed as a good predictor for maintenance of I-ADL among the rural community elderly in Japan: A 5-year follow-up study from TMIG-LISA. Geriatrics & Gerontology International, 3, S6–S14.

[bibr16-0733464820911542] Tolea M. I. Costa P. T. Terracciano A. Griswold M. Simonsick E. M. Najjar S. S. Scuteri A. Deiana B. Orrù M. Masala M. Uda M. Sclessinger D. Ferrucci L. (2010). Sex-specific correlates of walking speed in a wide age-ranged population. The Journals of Gerontology, Series B: Psychological Sciences & Social Sciences, 65(2), 174–184. 10.1093/geronb/gbp130 PMC2821942 20051464

[bibr17-0733464820911542] Tseng L. A. Delmonico M. J. Visser M. Boudreau R. M. Goodpaster B. H. Schwartz A. V. Simonsick E. M. Satterfield S. Haris T. Newman A. B. (2014). Body composition explains sex differential in physical performance among older adults. The Journals of Gerontology, Series A: Biological Sciences & Medical Sciences, 69(1), 93–100. 10.1093/gerona/glt027 PMC3859364 23682159

[bibr18-0733464820911542] Windham B. G. Griswold M. E. Wang W. Kucharska-Newton A. Demerath E. W. Gabriel K. P. Pompeii L. A. Butler K. Wagenknecht L. Kritchevsky S. Mosley T. H. (2017). The importance of mid-to-late-life body mass index trajectories on late-life gait speed. The Journals of Gerontology, Series A: Biological Sciences & Medical Sciences, 72(8), 1130–1136. 10.1093/gerona/glw200 PMC5861851 27811156

[bibr19-0733464820911542] World Health Organization. (2000). Obesity: Preventing and managing the global epidemic: Report of a WHO consultation (Electronic No. 894). http://www.who.int/nutrition/publications/obesity/WHO_TRS_894/en/11234459

